# Type I hypersensitivity in photoallergic contact dermatitis

**DOI:** 10.1016/j.jdcr.2023.11.032

**Published:** 2023-12-20

**Authors:** Shawn Afvari, Jonathan H. Zippin

**Affiliations:** a Department of Dermatology, Weill Cornell Medical College, New York, New York; b New York Medical College School of Medicine, Valhalla, New York

**Keywords:** allergic contact dermatitis, contact dermatitis, dermatitis, methyl anthranilate, patch test, photoallergy, photopatch test, photosensitivity, solar urticaria, type I hypersensitivity, type IV hypersensitivity

## Introduction

Photosensitivity represents a heterogenous group of diseases ranging in pathophysiology with subtypes clinically distinguished by phototesting. Testing involves measurement of a minimal erythemal dose to ultraviolet A (UV-A), ultraviolet B (UV-B), and visible light. Immediate reaction to light alone is deemed solar urticaria, a type I hypersensitivity reaction (HSR) to a UV-activated endogenous compound within the dermis or serum.^1^^,^^2^ After light testing, photo-allergens are exposed to the back for 24 hours and then irradiated with UV-A. Photoallergic contact dermatitis (PACD) is normally defined as a delayed-type (type IV) HSR upon contact with an exogenous compound, a prohapten, that is activated by UV exposure.^3^ In the present manuscript, we present 2 unique cases of PACD presenting as a type I HSR.

## Case 1

**Fig 1:**
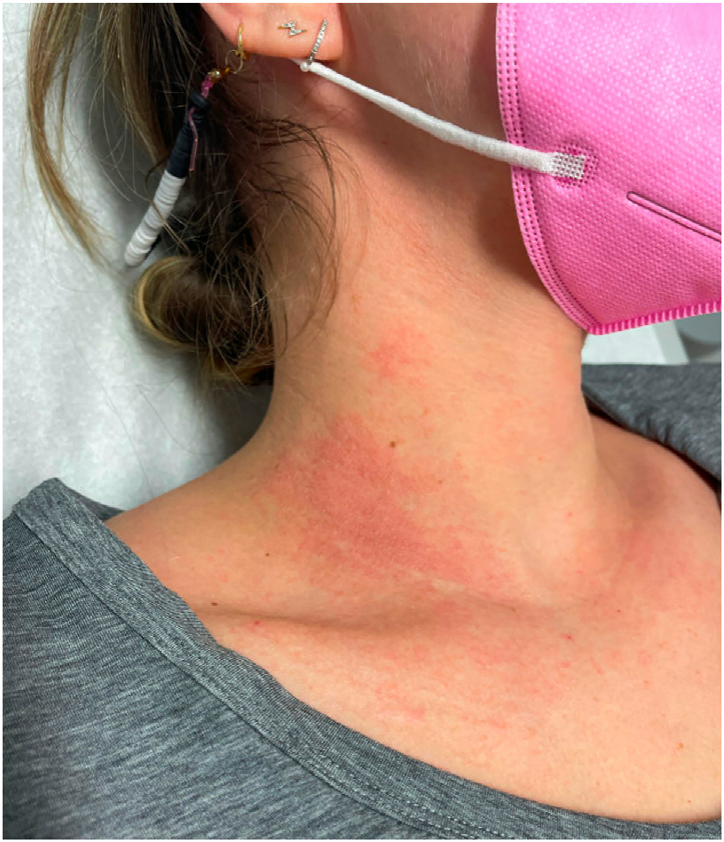
Initial patient presentation in case 1 with erythematous papules and plaques right side of the neck.

A 31-year-old woman with history of atopic dermatitis presented to dermatology for a recurrent rash on her chest, neck, and bilateral dorsal aspect of the feet for 3 months. The rash was pruritic and tender and recurred only during her travels to Puerto Rico and Florida. She reported consistent sunscreen usage throughout the year with no changes in personal products. Patient-provided images showed erythematous patches and wheals on the neck and bilateral dorsal aspect of the feet. Physical examination revealed erythematous papules and plaques, some with scale in a photo-distributed pattern on the upper-mid chest and bilateral side of the neck (Fig 1). The clinical differential diagnosis included PACD, polymorphous light eruption (PMLE), and collagen vascular disease. A punch biopsy of the right side of the neck displayed eczematoid and delayed dermal HSR. Taken together, the histopathologic and clinical findings were consistent with a diagnosis of either PACD or PMLE, and the patient was referred for photo-patch testing.

**Fig 2:**
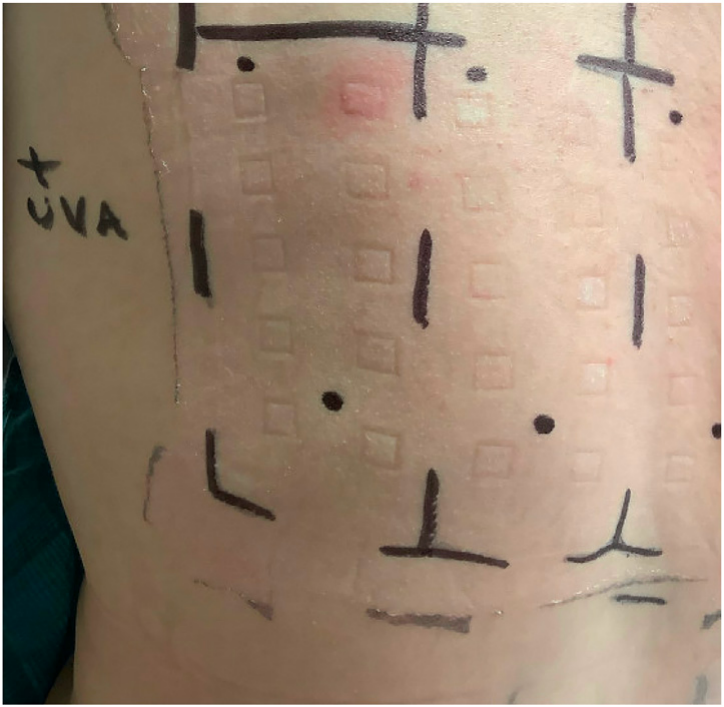
Immediate hypersensitivity reaction upon ultraviolet A-exposed methyl anthranilate patch in case 1.

Phototesting was performed using UV-B (5 spot-doses: 12–60 mJ/cm^2^), UV-A (5 spot-doses: 2–10 J/cm^2^), and 15 minutes of visible light. No immediate reaction (up to 3 hours) was visible for any light source. Two sets of 32 patches were applied to the back covering the Weill Cornell photo-allergen series. Twenty-four hours later, UV-A and visible light tests were negative at all doses, and UV-B was positive at 36 mJ/cm^2^. One set of photo-allergens was exposed to 10 J/cm^2^ of UV-A. An urticarial reaction to methyl anthranilate (MA) was noted only immediately after UV-A exposure (Fig 2). This reaction subsided over the remainder of the day. Subsequent visits during the next 3 days did not reveal persistent reaction to MA or evidence of delayed hypersensitivity among any other patch tests. The patient was educated regarding type I hypersensitivity and provided a safe-product list. No additional reactions have occurred with avoidance and antihistamine use.

## Case 2

A 48-year-old woman with history of asthma presented to dermatology for evaluation of a facial rash. A similar rash had recently erupted on other body areas often within minutes of sun exposure but rarely lasting longer than a day. She reported the facial rash was highly pruritic and characterized by coarse texture. She reported use of new over-the-counter topical products and spending more time outdoors. She was prescribed topical triamcinolone, oral prednisone and hydroxyzine, and the rash resolved in 10 days. Patient-provided images revealed well-demarcated erythema with possible pseudovesicles. Physical examination revealed residual xerosis overlying areas of past erythema on the face. The clinical differential diagnosis included solar urticaria, PACD, and PMLE.

Phototesting and photo-patch testing was performed through an identical protocol as in case 1. No immediate reaction was visible for any light source. UV-A and visible light tests were negative at all doses and UV-B was positive at 60 mJ/cm^2^. An immediate urticarial reaction was noted to MA only upon UV-A exposure and resolved over the day. Clinical visits 48 hours after UV-A exposure revealed a delayed reaction to MA consistent with a type IV hypersensitivity. The patient was educated regarding both type I and type IV HSR and provided a safe-product list.

## Discussion

Type IV HSR involves allergen binding to an antigen-presenting-cell in the epidermal or dermal layers followed by migration to lymph nodes and induction of a delayed clonal T-cell expansion. On the skin, this manifests as an eczematous reaction with erythema and edema commonly leading to scale. Type I cutaneous hypersensitivity, on the other hand, is a histamine-mediated reaction, presenting as erythematous wheals and urticaria. Although PACD has traditionally been defined as a type IV HSR, our cases clearly demonstrate type I hypersensitivity pathophysiology. To our knowledge, the only other case report of immediate photosensitivity to an exogenous compound was published in 1975; however, this type I HSR to chlorpromazine coexisted with an allergic contact dermatitis and delayed-type PACD to the compound.^4^

MA is a precursor to meradimate, which is approved by the United States Food and Drug Administration as a sunscreen and fragrance additive.^5^ The molecule is generally photostable due to intramolecular hydrogen bonding; however, it can degrade upon UV exposure.^6^ This mirrors an observed occurrence in various UV filters, where exposure to UV radiation can lead to degradation, possibly causing irritant or allergic responses. To our knowledge, there are no cases of type I (immediate) or type IV (delayed) hypersensitivity to MA that exist in the literature.

As a precursor to sunscreens and possible contaminant, our patients likely experienced this HSR upon application of their sunscreen products during summer months or travel to areas with a higher UV index than their baseline in the northeast. Given the clinical presentation and histopathologic findings in case 1, we suggest this immediate hypersensitivity may have served as an inciting reaction for development of a concomitant PMLE. This may explain how the rash persists and ranges from urticarial to erythematous papules and plaques. In case 2, the patient has both an immediate and delayed hypersensitivity to MA, which explains how the rash erupts immediately following sun exposure but persists with evolving morphology.

These cases serve to inform dermatologists of the possibility of a type I hypersensitivity PACD. It also highlights how testing for chemical intermediates in photo-allergy testing, akin to the testing of dimethylaminopropylamine in standard type IV testing, may uncover sensitization. We hope these cases will demonstrate the importance of immediate visualization after UV-A exposure during photo-allergy testing.

## Conflicts of interest

None disclosed.
